# Effect of Substituent Location on the Relationship between the Transition Dipole Moments, Difference Static Dipole, and Hydrophobicity in Squaraine Dyes for Quantum Information Devices

**DOI:** 10.3390/molecules28052163

**Published:** 2023-02-25

**Authors:** Maia Ketteridge, Austin Biaggne, Ryan Rau, German Barcenas, Olga A. Mass, William B. Knowlton, Bernard Yurke, Lan Li

**Affiliations:** 1 Micron School of Materials Science and Engineering, Boise State University, Boise, ID 83725, USA; 2 Department of Electrical and Computer Engineering, Boise State University, Boise, ID 83725, USA; 3 Center for Advanced Energy Studies, Idaho Falls, ID 83401, USA

**Keywords:** squaraine dye, substitution, density functional theory, exciton, optical properties

## Abstract

Aggregates of organic dyes that exhibit excitonic coupling have a wide array of applications, including medical imaging, organic photovoltaics, and quantum information devices. The optical properties of a dye monomer, as a basis of dye aggregate, can be modified to strengthen excitonic coupling. Squaraine (SQ) dyes are attractive for those applications due to their strong absorbance peak in the visible range. While the effects of substituent types on the optical properties of SQ dyes have been previously examined, the effects of various substituent locations have not yet been investigated. In this study, density functional theory (DFT) and time-dependent density functional theory (TD-DFT) were used to investigate the relationships between SQ substituent location and several key properties of the performance of dye aggregate systems, namely, difference static dipole (Δd), transition dipole moment (μ), hydrophobicity, and the angle (θ) between Δd and μ. We found that attaching substituents along the long axis of the dye could increase μ while placement off the long axis was shown to increase Δd and reduce θ. The reduction in θ is largely due to a change in the direction of Δd as the direction of μ is not significantly affected by substituent position. Hydrophobicity decreases when electron-donating substituents are located close to the nitrogen of the indolenine ring. These results provide insight into the structure–property relationships of SQ dyes and guide the design of dye monomers for aggregate systems with desired properties and performance.

## 1. Introduction

Organic dyes have been shown to aggregate in both natural and artificial systems [1,2,3,4]. When aggregated, the excited state wavefunctions of the dyes mix, resulting in a coherent sharing of excitation energy across multiple dyes (i.e., exciton delocalization). The unique excitonic coupling of dye-aggregates is crucial for applications used in organic photovoltaics [5,6,7,8], medical imaging [9,10,11,12,13], light harvesting [14,15,16,17], and quantum information science (QIS) [18,19,20,21]. Kasha and Davydov described excitonic coupling as the dipole–dipole coupling of adjacent dyes [22,23]. This coupling is further described by the Frenkel Hamiltonian, which is a similar form to the Hamiltonian describing multiparticle walks—a key aspect of quantum computing [24]. Therefore, molecular excitonic systems are particularly applicable to QIS applications [20,21]. From the Frenkel Hamiltonian, two parameters of interest emerge: the exciton hopping parameter (J_m,n_) and biexciton interaction energy (K_m,n_). The maximization of these parameters is key to the design of dye aggregates. J_m,n_ is associated with the transfer of an exciton between dyes, allowing for the delocalization of an exciton across two or more dyes. Assuming the dipole approximation, the J_m,n_ of a dimer is dependent on the transition dipole (μ) of the dye monomers, the cube of distance between dyes (R_m,n_), and relative dye orientations. The excitonic interaction energy, K_m,n_, which describes the interaction energy between two excitons, is dependent on the difference in the static electronic dipole between the excited and ground states (Equation (1) in the Methods section), referred to as difference static dipole (Δd). K_m,n_ allows for the creation of multiparticle entangled states. To perform quantum operations and achieve entanglement between excitons, J_m,n_ and K_m,n_ must be large. Hence, it is crucial to understand of how molecular structure influences Δd and μ as these properties must be maximized to allow for efficient quantum computation [21].

Free dyes in a concentrated solution can spontaneously form molecular aggregates [23,25,26]. However, the size and orientation of aggregated dyes can vary. There is significant interest in the methods of promoting aggregation that allow for the control of the number and orientations of aggregated dyes. DNA-templating is a particularly attractive method due to the ease of synthesis, conformational predictability of DNA complexes, and ability to promote dye aggregation [1,27,28,29,30,31,32,33]. DNA has a negligible effect on the electronic and spectroscopic properties of organic dyes, allowing for effective templating with minor interaction [34,35]. Other methods include the covalent linking of multiple dyes [36] and protein arrangement in natural light-harvesting systems [2]. Hydrophobicity has been shown to increase the J_m,n_ of organic dyes in DNA-templated aggregate systems, likely by bringing the dyes closer together [37]. Hydrophobicity can be increased through substitution with hydrophobic groups [38]. Modification of the Δd, μ, and hydrophobicity of dye monomers is key to the design and control of dye-aggregate systems for desired applications.

The angle between Δd and μ, termed here θ, could possibly be another important parameter in the design of QIS excitonic systems [18,19,24]. Dye molecules with orthogonal Δd and μ (θ = 90°) could be utilized to detect the presence of excitons on an adjacent dye. The dyes are arranged so that the μ values of the two dyes are orthogonal, canceling out the transition–dipole interactions. Therefore, the measured spectral shift is different if an exciton is located on one of the dyes.

**Figure 1:**
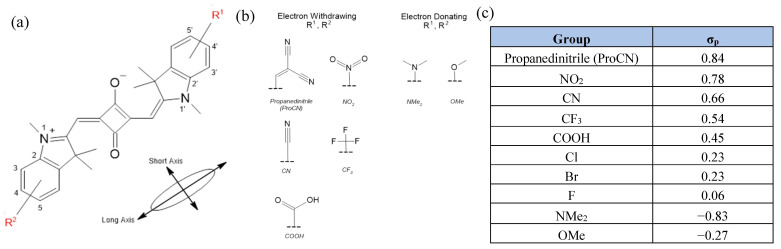
(**a**) Base molecular structure of an indolenine-based squaraine (SQ), with relevant substituent locations labeled. Electron-withdrawing groups (EWGs) are located on positions 3, 4, or 5, and electron-donating groups (EDGs) are located on the positions 3′, 4′, and 5′. (**b**) Chemical structures of the substituents used in this study, and (**c**) table of Hammett constants for all substituents, where negative Hammett constant indicates electron donating character and a positive Hammett constant indicates electron withdrawing character. [48,49].

Cyanines (Cys), such as Cy5, and squaraines (SQs) are advantageous choices for dye-aggregates due to their strong absorbance peak in the visible range with a minimum vibronic shoulder [39]. In addition, SQs exhibit resistance to photobleaching [40] and their chemical structures can be easily modified [41,42]. Density functional theory (DFT) and time-dependent (TD-) DFT have been shown to be very useful in predicting and optimizing the optical and electronic properties of Cy, SQ, and dyes with a similar structure [38,41,43,44,45,46,47]. Using DFT and TD-DFT, we computationally identified a set of design principles for the dye monomers in excitonic systems [38,46]. In particular, the substitution of the SQ or Cy indolenine rings was found to have a small effect on μ while significantly enhancing Δd. In the substituted SQs, the absolute value of the Hammett constant of a substituent was determined to be positively correlated with both μ and Δd [46]. The Hammett constant could be used to quantify the strength a substituent’s electron-donating or electron-withdrawing character [48]. The symmetric substitution of Cy dyes (e.g., Cy5) with electron-withdrawing substituents resulted in greater Δd enhancement than electron-donating groups. Asymmetric substitution of Cy5 and SQ with electron-donating and withdrawing groups on opposite sides of the molecule (“push-pull” dyes) also resulted in high Δd values [38]. The Δd values of substituted SQ have been computationally found in a range of 0 D–3.20 D, while μ has ranges from 14.2 to 16.3 D [46]. Computational studies of excitonic properties have only investigated the effect of SQ and Cy5 substitution at a single site (position 5, Figure 1a). However, substitution at other locations is possible, and location may have significant effects on our properties of interest.

Following our previous studies [38,46], we investigated the effect of the substituent position on the optical properties of indolenine SQs to develop additional dye monomer design principles for QIS excitonic applications. Two electron-donating groups (EDGs) and eight electron-withdrawing groups (EWGs) (Figure 1b,c) were selected based on their performance in obtaining favorable excitonic properties in our previous studies [46]. The EDGs and EWGs were placed on opposite sides of an SQ dye, and the positions of the substituents was varied (Figure 1a). In addition, the EDGs and EWGs were placed on the same side of the SQ molecule, leaving the other side unsubstituted. Using DFT and TD-DFT, we examined the effects of positioning when the substituents were in close proximity. The values of Δd, μ, the angle between Δd and μ (termed here, θ), and the logarithm of the water/*n*-octanol partition coefficient logP used for quantifying hydrophobicity were calculated for each position variation to ensure that the dyes remained overall hydrophobic. We found that substituents at additional positions allowed for more fine control of dye properties. We also identified factors influencing θ.

## 2. Results

### 2.1. Squaraine with Substituents on Opposite Sides (“Push-Pull” Dyes)

**Figure 2:**
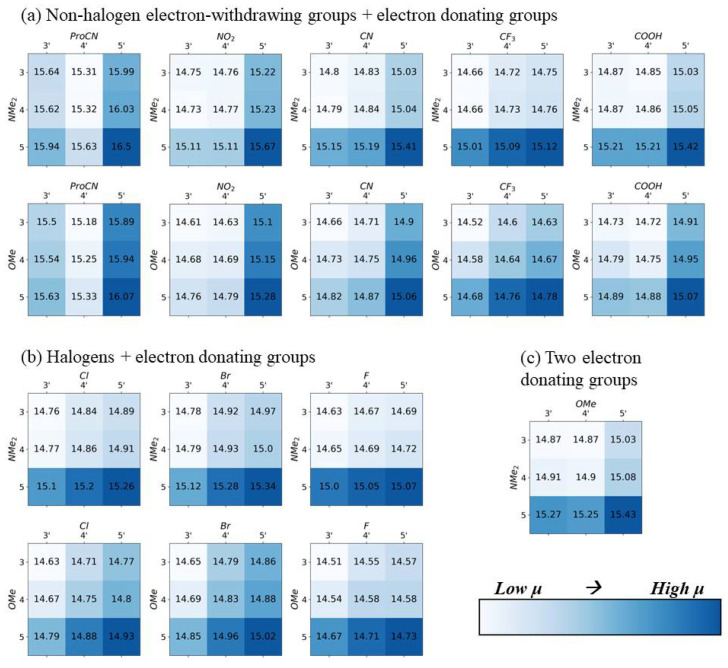
DFT-calculated transition dipole moments (µ), in Debye, for all opposite-side substituent pairs. Darker blue indicates a larger transition dipole moment. All calculations were performed with implicit solvation in water, using the integral equation formalism polarizable continuum model (IEFPCM) method (see more details in the methods section). Darker colors indicate higher values. The color range is only consistent for each substituent pair, and color-coding should not be used to compare values between pairs.

**Figure 3:**
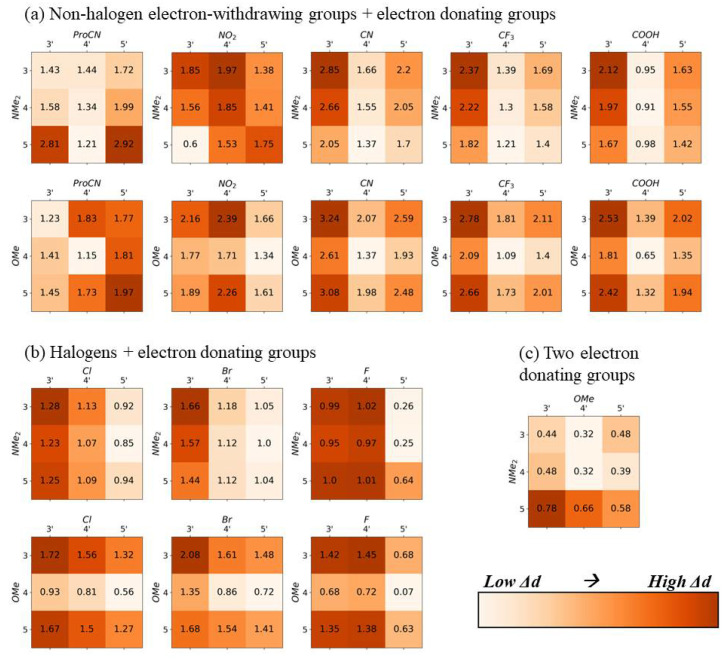
DFT-calculated difference static dipole moments (Δd), in Debye, for all opposite-side substituent pairs. Darker orange indicates a larger static dipole difference. All calculations were performed with implicit solvation in water, using the integral equation formalism polarizable continuum model (IEFPCM) method (see more details in the methods section). Darker colors indicate higher values. The color range is only consistent for each substituent pair, and color-coding should not be used to compare values between pairs.

**Figure 4:**
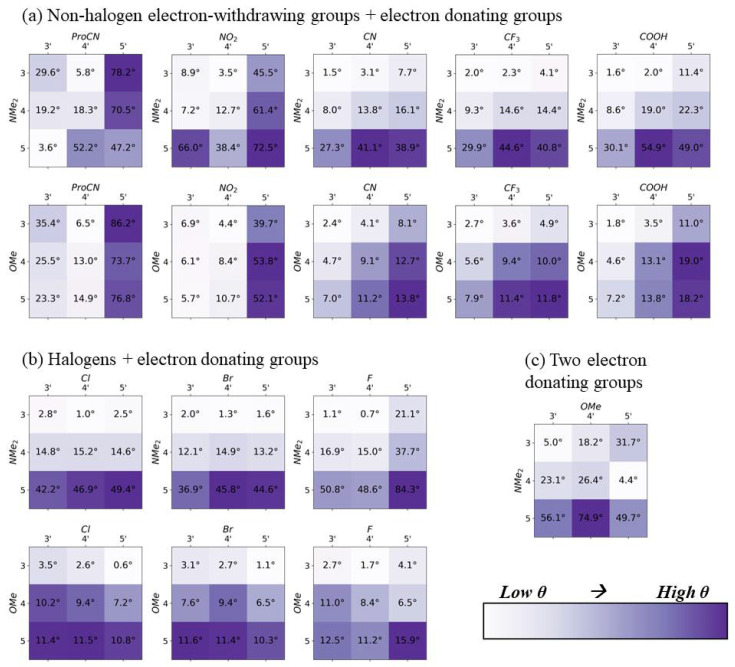
DFT calculated angle θ values between Δd and μ, in degrees, for all opposite-side substituent pairs. Darker purple indicates a larger θ. All calculations were performed with implicit solvation in water, using the integral equation formalism polarizable continuum model (IEFPCM) method (see more details in the methods section). Darker colors indicate higher values. The color range is only consistent for each substituent pair, and color-coding should not be used to compare values between pairs.

**Figure 5:**
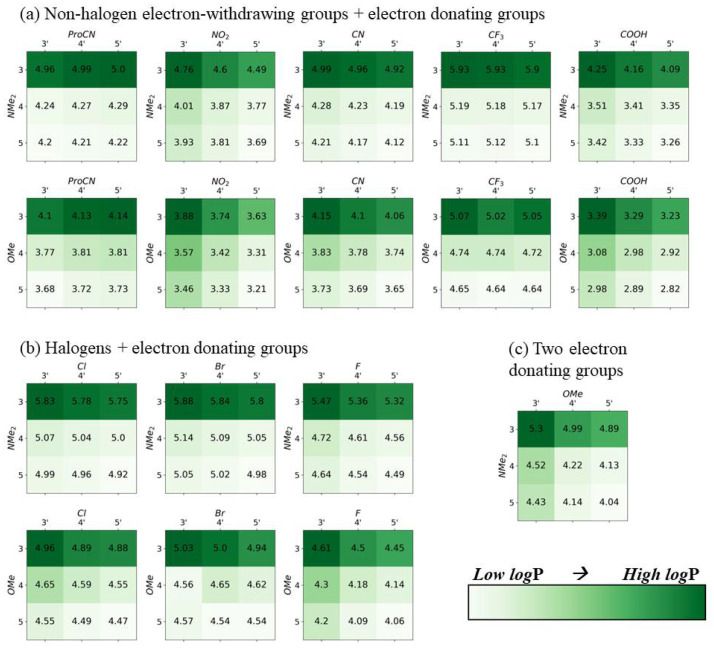
DFT-calculated logarithm of water/*n*-octanol partition coefficient logP, used for quantifying hydrophobicity for all opposite-side substituent pairs. Darker green indicates a larger logP. The ground-state geometry, optimized in vacuum, was used for all calculations. Darker colors indicate higher values. The color range is only consistent for each substituent pair, and color-coding should not be used to compare values between pairs.

In Figure 2, Figure 3, Figure 4 and Figure 5, the electron-withdrawing group positions are located on the vertical axis, and the EDGs are located on the horizontal axis (see Figure 1a for definitions of position). The group combination OMe–NMe_2_ is an exception, with the EDGs placed on both ends of the dye. To emphasize trends in substituent location, the color scale in Figure 2, Figure 3, Figure 4 and Figure 5 is scaled to the range of values found for each substituent pair and cannot be used to compare between substituent pairs. See Figures S1–S4 for versions of these figures with a global color scale.

First, we investigated the effect of substituent chemical structure and location on the magnitude of the transition dipole moment (μ) and static dipole difference (Δd). Aggregates of dye monomers with higher μ results in stronger excitonic coupling [22,50]. For all opposite-side substituent combinations, configurations with substituents closer to position 5 or 5′ result in a significantly higher magnitude μ compared to the dyes with substituents on positions 3 or 3′ (Figure 2). This is consistent with the results from other dyes, where modifying the molecule along the transition dipole axis increases the value of the μ [51]. The transition dipole of indolenine SQ tends to align with the dye’s long axis. This trend is consistent in both vacuum and implicit water. Solvation in water tends to increase all transition dipole magnitudes by approximately 1 Debye (Figure S5). The substituent combination with the highest transition dipole is SQ-ProCN-NMe_2_, which is consistent with previous results that suggested that substituents with higher magnitude Hammett constants have a greater effect on transition dipole [46]. In both water and vacuum, the difference between the maximum and minimum μ for a single substituent pair ranges between ~0.5 and 1.0 Debye, which is ~3–7% difference (Figure S5).

The largest difference static dipole (Δd) tends to occur when one or both substituents are placed on positions 3 or 3′, furthest from the main axis of the dye (Figure 3), except for EWGs ProCN and F, which have the greatest and smallest Hammett constants of all EWGs in this study, respectively. This trend is not as consistent as the trend observed in the transition dipole. The trend in the difference static dipole is most likely due to an increase in molecule asymmetry. Unsubstituted SQ is symmetrical around the long axis; therefore, placing a substituent off the long axis increases asymmetry. This trend is more pronounced in vacuum than in implicit water (Figure S6). The difference between maximum and minimum Δd for a single substituent also ranges ~0.5–1 Debye, which often results in a two-fold increase from the minimum to the maximum value of a single substituent pair. Δd can be greatly increased by changing the location of the substituent. For dyes with the 5,5′- substitution, greater absolute value Hammett constants result in larger Δd, which is consistent with our previous studies [46]. However, this trend is not observed when the largest Δd in each subsistent pair is selected.

We anticipate that the angle between Δd and μ (θ) will be critical in the future development of QIS molecular excitonic systems [18,24]. As such, we investigated the relationship between θ and substituent location. Within an opposite-side substituent pair, the angle θ is greatest when at least one substituent is located on the positions 5 or 5′ (Figure 4). With reference to the dye’s long axis, the direction of μ is not greatly affected by substitution. Therefore, the changes in θ are largely due to changes in the direction of Δd. Placing substituents off the main axis of the dye tends to align the Δd along the long axis of SQ, therefore reducing θ. In general, θ is larger in water than in a vacuum (Figure S7). The substituent Hammett constant has no apparent relationship to θ.

Increased hydrophobicity has been shown to increase excitonic coupling [37]. Hydrophobicity, quantified with logP using Equations (2) and (3) in the Methods section, increases if the EWG is located on positions 3′ (See Figure 5). The ground-state geometry, optimized in the solvent of interest, was used for all solvated calculations. In the presence of two EDGs, NMe_2_ on positions 3′ results in a larger increase in logP than OMe on position 3. The increase is small, typically less than 1 unit. For the structure optimized in the solvent of interest, unusually large increases of 3–4 units are observed with NMe_2_ on position 3′ (see Figure S8). This large increase is caused by the NMe_2_ rotating out of plane in the water-solvated structure while lying in-plane in the vacuum and n-octanol-solvated structure. The relationship between hydrophobicity and substituent positions is consistent between both calculation procedures, with the only difference being the magnitude of the effect.

### 2.2. Squaraine with Substituents on the Same Side

**Figure 6:**
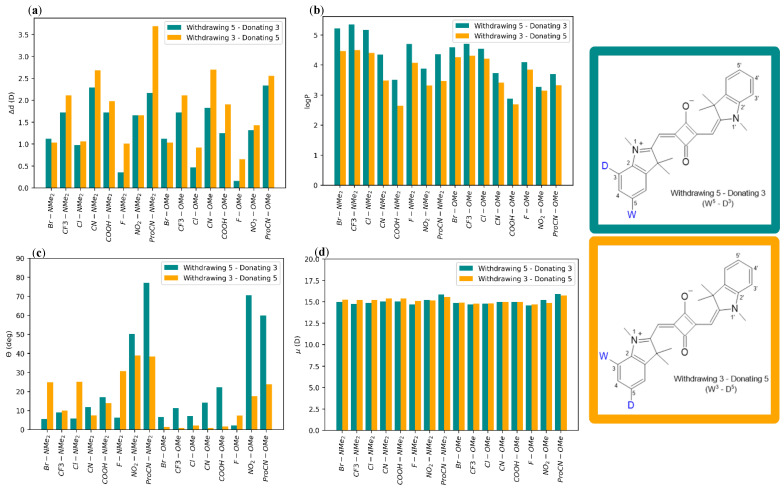
(**a**) Δd values of same-side substituent pairs, (**b**) logP of same-side substituent pairs, (**c**) θ values for all same-side substituent pairs, and (**d**) μ values of same-side substituent pairs. “Withdrawing 5–Donating 3” refers to a configuration where the first listed substituent is located on position 5, and the second listed substituent is located on position 3. “Withdrawing 3–Donating 5” refers to a configuration where the first listed substituent is on position 3 and the second listed substituent is placed on position 5. Δd, θ, and μ calculations were performed in implicit water.

To investigate the effect of placing the EDG and EWG in close proximity, we placed substituents on one side of the dye, leaving the opposite side unsubstituted. A substitution configuration W^5^–D^3^ refers to a dye wherein an electron-withdrawing group is located on position 5 and an electron-donating group is located on position 3 (see Figure 1a for position numbering). W^3^–D^5^ refers to the reverse substitution pattern. These positions were selected due to their synthetic accessibility. Overall trends in μ magnitude are consistent in both the implicit water and vacuum (Figure S9). For dyes with an electron-donating NMe_2_ group, the μ is consistently higher by (<1 D) in the W^3^–D^5^ configuration than in the W^5^–D^3^ configuration (Figure 6d). Conversely, this is not observed for electron-withdrawing NO_2_ or ProCN groups, even though the Hammett constants of these groups are comparable in magnitude to the Hammett constant of donating NMe_2_. There is no clear trend between the substituent position and μ when OMe is used as the electron-donating group.

Substituent configuration could affect Δd. Particularly, the W^5^–D^3^ configuration results in a larger Δd than the W^3^–D^5^ configuration for all but two substituent combinations. These exceptions are Br–OMe, showing a greater Δd in the W^3^–D^5^ than in the W^5^–D^3^ configuration, and NO_2_–NMe_2_, showing no difference between the W^5^–D^3^ and W^3^–D^5^ configurations (Figure 6a). This trend is consistent in vacuum and implicit water (Figure S10). The largest Δd is observed for SQ-ProCN-NMe_2_ in the W^5^–D^3^ configuration. This finding fits with previously identified trends, as ProCN and NMe_2_ are the strongest EWGs and EDGs, respectively.

Next, we proceeded with the evaluation of dye hydrophobicity by calculating logP using Equations (2) and (3) in the Methods section. We found that, regardless of substituent choice, logP is larger for the position 5–position 3 configuration compared to the position 3–position 5 configuration (Figure 6b). This trend is consistent with the results of the “push-pull” dyes, where placing an EDG on position 3 greatly increases logP.

In dyes containing OMe, the position 5–position 3 configuration generally results in a larger θ. In this configuration, the EDG is located along the long axis of the dye. The dyes containing NMe_2_ show no clear trend (Figure 6c and Figure S11).

## 3. Discussion

Maximizing Δd and μ is crucial when designing dyes for QIS excitonic aggregate applications. Although placing substituents along the long axis of the dye results in the largest increases in μ magnitude (as seen in Figure 2), the placement of a substituent off the long axis results in larger increases in Δd magnitude (as seen in Figure 3). Proportionally, the effect of the substituent position on Δd is much larger than its effect on μ. A two-fold increase in Δd could be achieved through the modification of the substituent positions alone; however, μ could only be increased by ~3–7%. These increases in Δd magnitude usually correspond to a rotation of Δd direction towards the long axis of the dye, as shown in Figure 4. In agreement with our previous studies [38,46], substituents with the larger absolute values of Hammett constants result in an increase in the magnitudes of μ. Although the larger absolute values of Hammett constants are correlated with the higher Δd magnitudes when both substituents are placed on the 5 and 5′ respectively, this trend does not hold for any the other substituent locations.

We hypothesize that the angle between Δd and μ (θ) would be key in the design of complex dye aggregate systems. Our results also suggest an inverse correlation between Δd magnitude and θ. As the magnitude of Δd increases, the maximum observed θ value decreases, suggesting that large Δd values are often along the same axis as μ in indolenine SQs. For SQ, μ tends to be located along the long axis of the dye, and substitution has little effect on its direction. However, substituent location has a large effect on the Δd direction, resulting in rotations of up to 85 degrees with reference to the dye’s long axis.

Our results suggest a general strategy for the modification of dye optical properties via substitution. If a “push-pull” SQ with a large Δd that is aligned along the long axis of the dye is desired, substituents should ideally be placed off the main dye axis. If a Δd that is orthogonal to μ is desired, Δd should be placed as close as possible to the long axis of the dye. Substituents with the large absolute values of Hammett constants should be chosen to maximize μ. When Δd is placed on position 5, along the long axis of the dye, the high absolute value of Hammett constant also results in an increase in the magnitude of Δd; however, this trend does not hold for other positions. The direction of Δd is more influenced by the modification of the substitution location than the direction of μ. An interesting consequence of the influence of the substituent location on Δd and μ is that the direction of Δd can be modified with substituent change without greatly impacting the direction of μ, which is attractive in cases where one would like to change Δd without affecting μ.

For dyes with substituents on positions 3 and 5, interchanging the location of the electron-withdrawing and electron-donating groups does not significantly affect the magnitude of μ (Figure 6d and Figure S7). As shown in Figure 6a Δd is significantly affected by interchanging the locations, but a clear trend only emerges in dyes containing OMe, where placing the electron-donating group on position 5 increases Δd.

The chemical nature and location of the substituents affect not only the optical and electronic properties of dyes (such as μ and Δd) but can also affect dye hydrophobicity. In general, increased hydrophobicity has been experimentally shown to increase exciton hopping parameter J_m,n_ in DNA-templated dye aggregates [37]. To ensure that the chemical nature and locations of substituents do not greatly decrease overall dye hydrophobicity, we evaluated the relationship between substituent position and hydrophobicity via calculated logP. For “push-pull” dyes, placing the electron-donating group on position 3 or 3′ increases the calculated logP by less than one. This was observed when the ground-state optimized geometry in a vacuum was used for all solvated calculation. However, when the geometry was optimized in the solvent of interest, placing NMe_2_ on position 3′ could increase the calculated logP, a measure of hydrophobicity, by a factor of three to four. This was caused by NMe_2_ rotating out of plane in the water-solvated structure. Our results suggest that hydrophobicity is significantly affected by the location of EDGs but is not significantly impacted by the location of electron-withdrawing groups.

## 4. Methods

The Gaussian 16 [52] software package was used to perform all DFT and TD-DFT calculations. Initial dye structures were built using the GaussView GUI [53]. Calculations and optimizations were performed using the M062X hybrid functional [54] and the 6-31+g(d,p) basis set [55,56], which we previously identified as applicable for similar dyes [38,46]. Gaussian 16’s ultrafine integration grid was applied, and the ground-state geometry was optimized using Gaussian 16’s default settings. Vibrational frequency calculations were performed at the optimized ground-state geometry to verify that a true minimum was found.

Single point excited state calculations at the ground-state geometry were performed to determine the transition dipole and permanent dipole of the Frank–Condon first excited singlet state (S_0_–S_1_). The difference static dipole (Δd) was determined by calculating the difference between the excited state and ground state electronic dipole moments, using the following equation [57]:

(1) $$\Delta d=\sqrt{{\left(d_{x}^{ES}-d_{x}^{GS}\right)}^{2}+{\left(d_{y}^{ES}-d_{x}^{GS}\right)}^{2}+{\left(d_{z}^{ES}-d_{z}^{GS}\right)}^{2}}$$

where $d_{i}^{j}$ is the Cartesian component of the static dipole moment, j refers to the excited state (*ES*) or ground state (*GS*) dipole moment, and *i* refers to the *i*-th cartesian component.

To estimate the solvent effects on μ and Δd, implicit solvation with the integral equation formalism polarizable continuum model (IEFPCM) [58,59] was applied to ground-state optimization and excited-state, single-point calculations. As with similar studies [60,61,62], IEFPCM was used for the calculation of both excited and ground-state properties in solvent. The nonequilibrium solvation condition was applied for excited state calculations to model the excitation of dye molecules in solvent.

Hydrophobicity of similar indolenine SQ dyes has been experimentally shown to correspond to stronger excitonic coupling in DNA-templated aggregates. Computational prediction of hydrophobicity was comparable to experimental values [37]. For solvation energy calculations, the standardized mean difference (SMD) variation of the IEFPCM method [63] was used, which has been shown as a useful method for the solvation energy calculations of organic molecules [64]. Solvation energy ($\Delta G_{solv}$) was determined by subtracting the energy in vacuum from the energy in solvent according to the following equation [35,63]:

(2) $$\Delta G_{solv}=E_{solv}-E_{v}$$

where $E_{solv}$ is the ground-state energy in solvent, the $E_{v}$ is ground-state energy in vacuum. Two procedures to determine the $E_{v}$ were implemented. For the first method, the ground-state geometry in vacuum was used for solvated calculations in water and n-octanol. Alternatively, the geometry was optimized in the solvent of interest using the SMD variation of the IEFPCM method. The solvation energy in water and n-octanol were used to estimate the logarithm of the water/n-octanol partition coefficient (log*P_w_*_/*o*_) using the following equation [37,65].

(3) $$\log P_{w/o}=\frac{\Delta G_{o}^{{}^{\circ}}-\Delta G_{w}^{{}^{\circ}}}{2.313RT}$$

where R is the gas constant, *T* was considered at room temperature, $\Delta G_{0}^{{}^{\circ}}$ is the energy of solvation in n-octanol, and $\Delta G_{w}^{{}^{\circ}}$ is the energy of solvation in water.

Eight electron-withdrawing substituents and two electron-donating substituents were selected to sample a wide range of Hammett constants (See Figure 1a, b). In a previous work, our group found that substituents with larger Hammett constant magnitudes were correlated with larger effects on SQ’s μ and Δd [46]. Electron-donating and electron-withdrawing substituents were placed on opposite ends of SQ to create an electron “push-pull” type dye. The location of the electron-withdrawing group was varied between positions 3 (ortho), 4 (meta), and 5 (para), and the location of the electron-donating group was varied between position 3′ (ortho), 4′ (meta), and 5′ (para). Position 6 was not considered due to the absence of synthetic access to the substituent at this site of the indolenine ring. A total of nine “push-pull” position variations were considered for each pair of substituents. One donating–donating substituent pair (OMe–NMe_2_) was also tested using the same set-up as the “push-pull” dyes.

The effect of placing both electron-donating and electron-withdrawing groups on the same side of the dye was also investigated. The substituents were placed on positions 3 and 5, resulting in two same-side position variations for each substituent pair.

For the function of some QIS molecular excitonic systems, the relative angle between the difference static dipole and transition dipole (θ) is relevant. The cartesian components of μ and Δd were reported directly by Gaussian software, and the angle between the vectors was determined using the dot product formula. The transition dipole was treated as a double-headed vector for the purposes of calculating θ. Therefore, θ ranges from 0 to 90°.

## 5. Conclusions

We employed DFT and TD-DFT to study the effects of the substituent type and location on the difference static dipole (Δd), transition dipole (μ), angle between Δd and μ (θ), and hydrophobicity (i.e., logP) of indolenine SQ. Our work affirmed our previous studies suggesting that substituents with the large absolute values of Hammett constants result in a larger increase in Δd and μ. We identified additional design rules for the creation of dyes for QIS excitonic applications. Substituents should be placed along the long axis of the dye (i.e., positions 5 and 5′) to obtain the maximum μ increase while substituents should be placed off the long axis of the dye (positions 3 and 3′) to maximally increase Δd. “Push-Pull” dyes, where electron-withdrawing groups and EDGs are placed on opposite side of a dye, result in high Δd magnitudes. Placing substituents along the long axis of the dye (positions 5 and 5′) results in the highest increases inμ. These increases are small, generally between 3 and 7%. Conversely, placing substituents off the main axis of the dye (positions 3 and 3′) generally results in a two-fold increase in Δd, as well as a change in the direction of Δd to align more with the μ and the long axis of the dye. Subsistent locations have a large impact on the magnitude of Δd, which can lead to two-fold increases. If more orthogonal Δd and μ are desired, substituents should be placed along the long axis of the dyes. There is a trade-off between Δd magnitude and the angle between Δd and μ. Hydrophobicity is increased when an electron-donating group is placed close to the nitrogen of the indolenine ring. Our results reveal dye structure–property relationships and guide the design of dye monomers for aggregate systems with a desired performance and provide a framework, on which further studies in this area can be pursued.

## Data Availability

Select solvation energy, dipole moment, angle, and logP data presented in this study is available in the Supporting Information. All other data presented are available upon request from the corresponding author. We also plan to make all data available in a publicly accessible data repository in the near future.

## Supplementary Materials

The following supporting information can be downloaded at: https://www.mdpi.com/article/10.3390/molecules28052163/s1, Figure S1: Global-Scale Transition Dipoles; Figure S2: Global-Scale Difference Static Dipoles; Figure S3: Global-Scale θ Values; Figure S4: Global Scale logP; Figure S5: Vacuum Transition Dipoles; Figure S6: Vacuum Difference Static Dipoles; Figure S7: Vacuum θ values; Figure S8: Additional logP values; Figure S9: Same-side vacuum transition dipoles; Figure S10: Same-side vacuum difference static dipoles; Figure S11: Same-side θ values.

## Sample Availability

Samples of the compounds are not available from the authors.
