# Supplementary material for: Effect of Substituent Location on the Relationship between the Transition Dipole Moments, Difference Static Dipole, and Hydrophobicity in Squaraine Dyes for Quantum Information Devices
Source: Molecules. 2023 Feb 25;28(5):2163. doi: 10.3390/molecules28052163 (PMC10004711; doi:10.3390/molecules28052163)
Supplement: Supplementary file 1 [file molecules-28-02163-s001.zip › molecules-2177902-supplementary.pdf]

Supplemental Information:

(A)

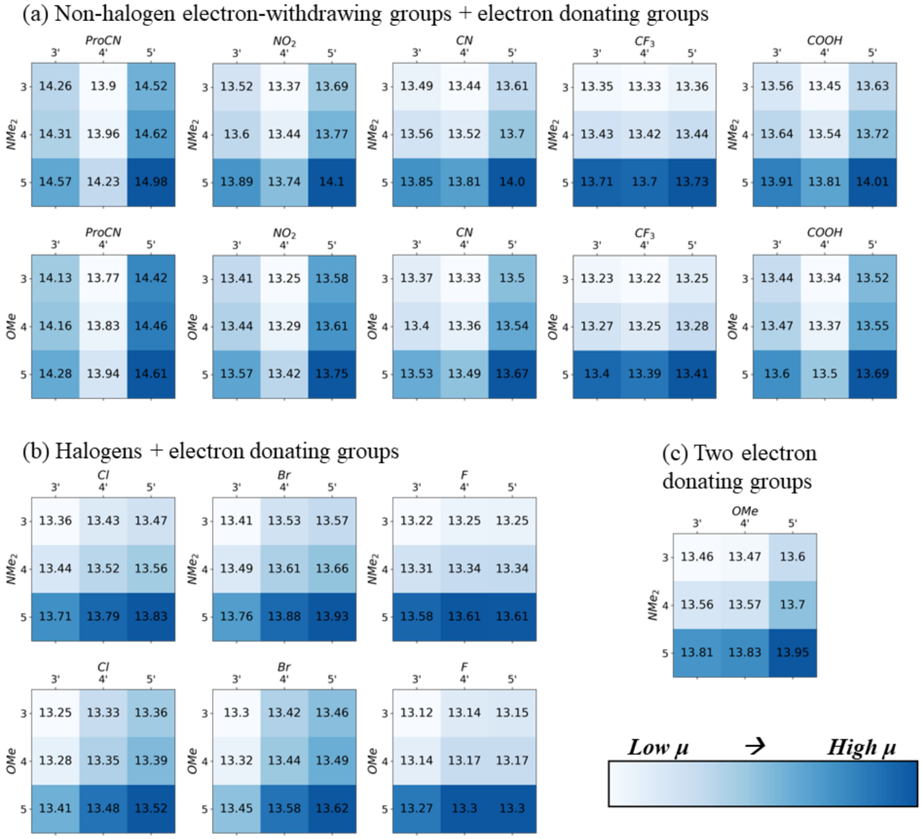

(B)

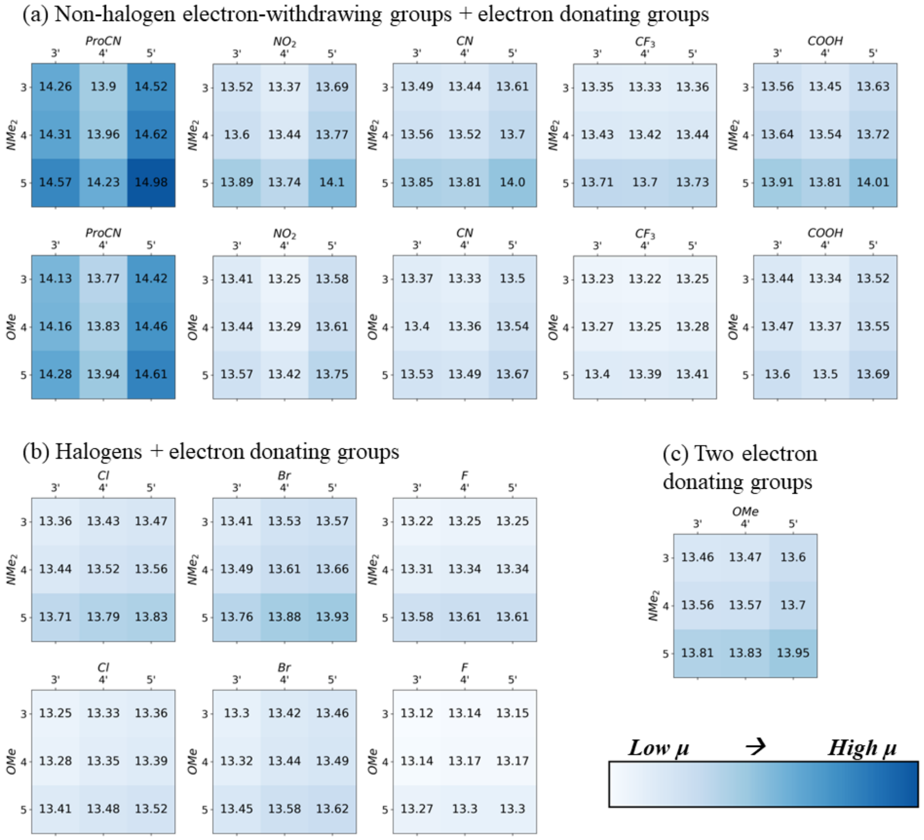

Figure S1: Density functional theory (DFT) – calculated transition dipole moments (μ), in Debye, for all opposite-side substituent pairs. Darker blue indicates a larger magnitude μ. All calculations were done in vacuum. Darker colors indicate higher values. (A) – The color range is scaled for each subsistent pair. (B) The color scale is global, and color can be used to compare between subsistent pairs.

(A)

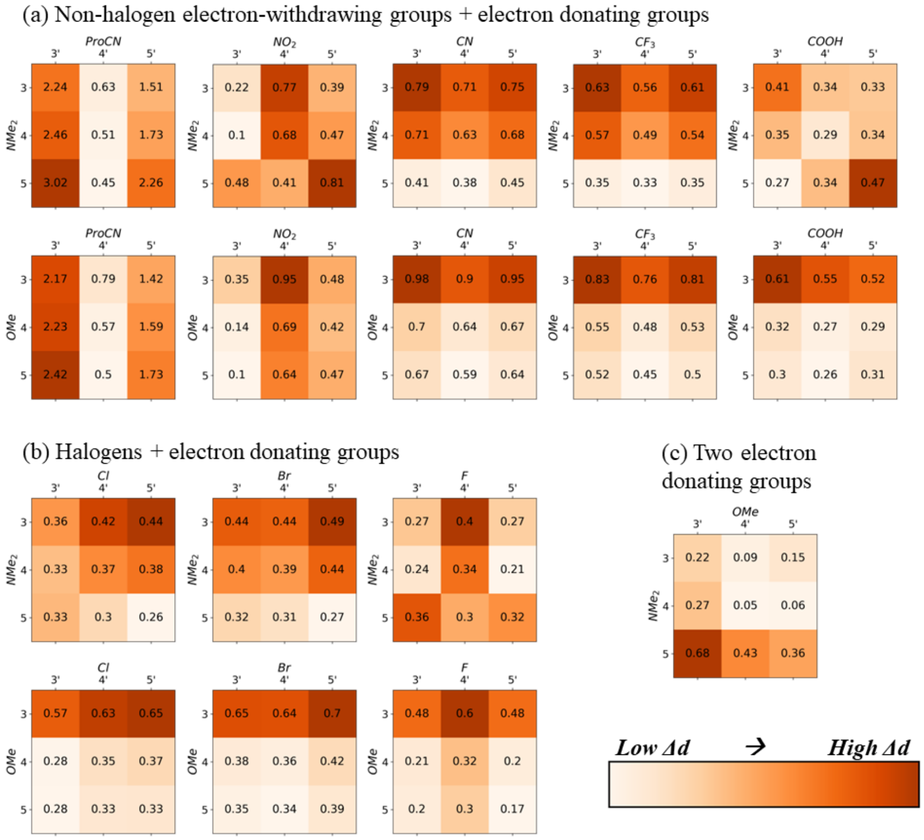
(a) Non-halogen electron-withdrawing groups + electron donating groups
ProCN
NMe2
2.24 0.63 1.51
2.46 0.51 1.73
3.02 0.45 2.26
NO2
NMe2
0.22 0.77 0.39
0.1 0.68 0.47
0.48 0.41 0.81
CN
NMe2
0.79 0.71 0.75
0.71 0.63 0.68
0.41 0.38 0.45
CF3
NMe2
0.63 0.56 0.61
0.57 0.49 0.54
0.35 0.33 0.35
COOH
NMe2
0.41 0.34 0.33
0.35 0.29 0.34
0.27 0.34 0.47
ProCN
OMe
2.17 0.79 1.42
2.23 0.57 1.59
2.42 0.5 1.73
NO2
OMe
0.35 0.95 0.48
0.14 0.69 0.42
0.1 0.64 0.47
CN
OMe
0.98 0.9 0.95
0.7 0.64 0.67
0.67 0.59 0.64
CF3
OMe
0.83 0.76 0.81
0.55 0.48 0.53
0.52 0.45 0.5
COOH
OMe
0.61 0.55 0.52
0.32 0.27 0.29
0.3 0.26 0.31
(b) Halogens + electron donating groups
Cl
NMe2
0.36 0.42 0.44
0.33 0.37 0.38
0.33 0.3 0.26
Br
NMe2
0.44 0.44 0.49
0.4 0.39 0.44
0.32 0.31 0.27
F
NMe2
0.27 0.4 0.27
0.24 0.34 0.21
0.36 0.3 0.32
Cl
OMe
0.57 0.63 0.65
0.28 0.35 0.37
0.28 0.33 0.33
Br
OMe
0.65 0.64 0.7
0.38 0.36 0.42
0.35 0.34 0.39
F
OMe
0.48 0.6 0.48
0.21 0.32 0.2
0.2 0.3 0.17
(c) Two electron donating groups
OMe
NMe2
0.22 0.09 0.15
0.27 0.05 0.06
0.68 0.43 0.36
Low Δd → High Δd

(B)

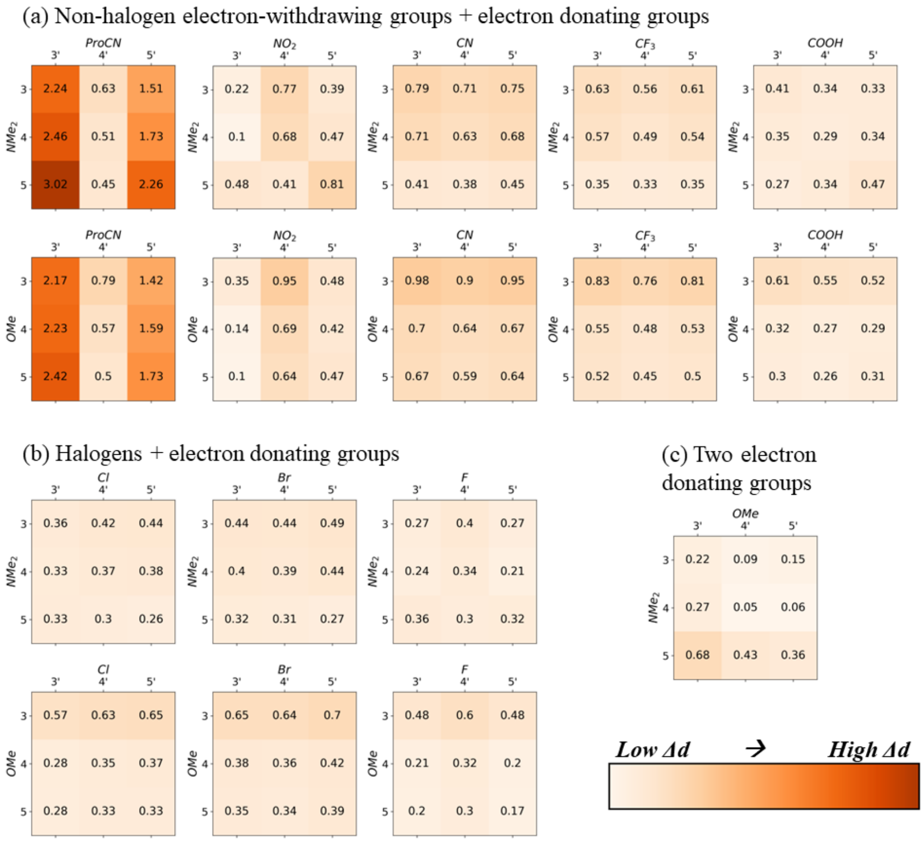

Figure S2: DFT - calculated difference static dipole (Δd), in Debye, for all opposite-side substituent pairs. Darker orange indicates a larger magnitude Δd. All calculations were done in vacuum. Darker colors indicate higher values. (A) - The color range is scaled for each subsistent pair. (B) The color scale is global, and color can be used to compare between subsistent pairs.

(A)

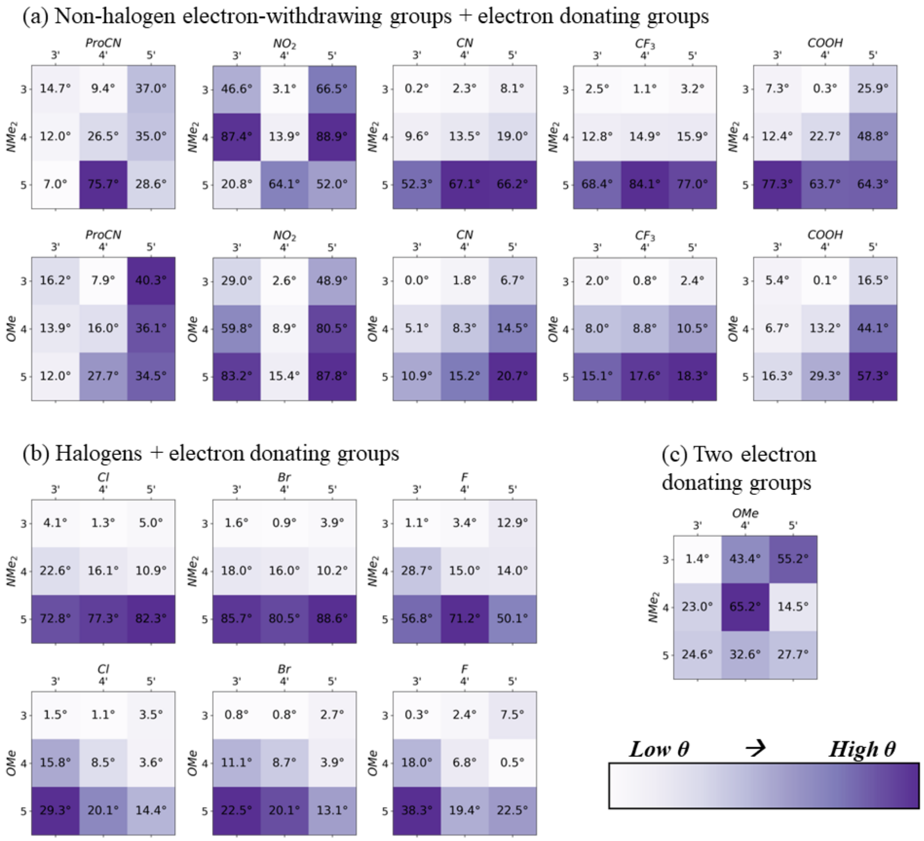
(a) Non-halogen electron-withdrawing groups + electron donating groups
ProCN
NO2
CN
CF3
COOH
NMe2
OMe
(b) Halogens + electron donating groups
Cl
Br
F
(c) Two electron donating groups
Low θ → High θ

(B)

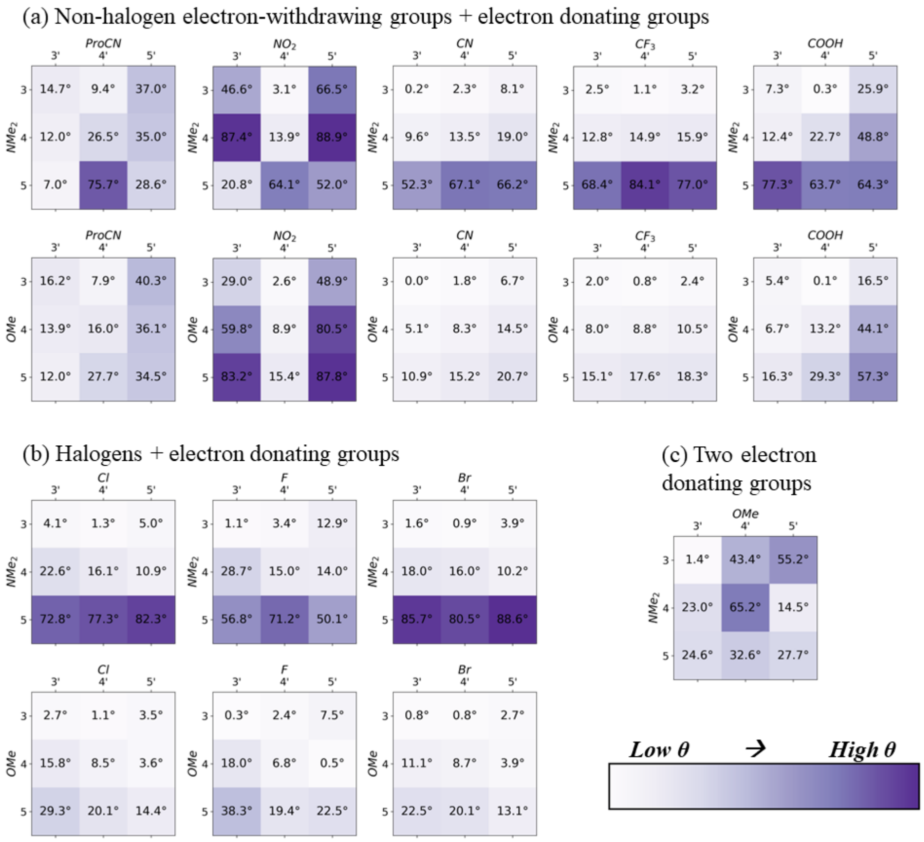

Figure S3: DFT-calculated θ *values between* μ *and* Δ*d, in degrees, for all opposite-side substituent pairs. Darker purple indicates a larger* θ. *All calculations were done in vacuum. Darker colors indicate higher values. (A) - The color range is scaled for each subsistent pair. (B) The color scale is global, and color can be used to compare between subsistent pairs.*

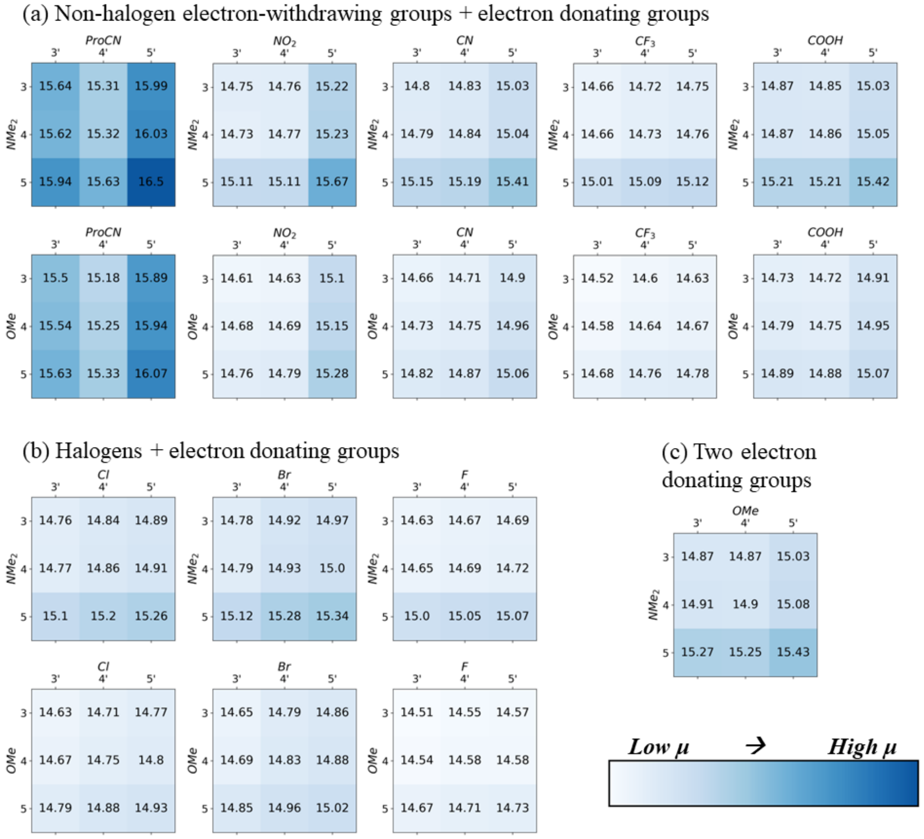

Figure S4: *DFT-calculated μ, in Debye, for all opposite-side substituent pairs. Darker blue indicates a larger magnitude μ. All calculations were done in implicit water. Darker colors indicate higher values. The color scale is global, and color can be used to compare between subsistent pairs.*

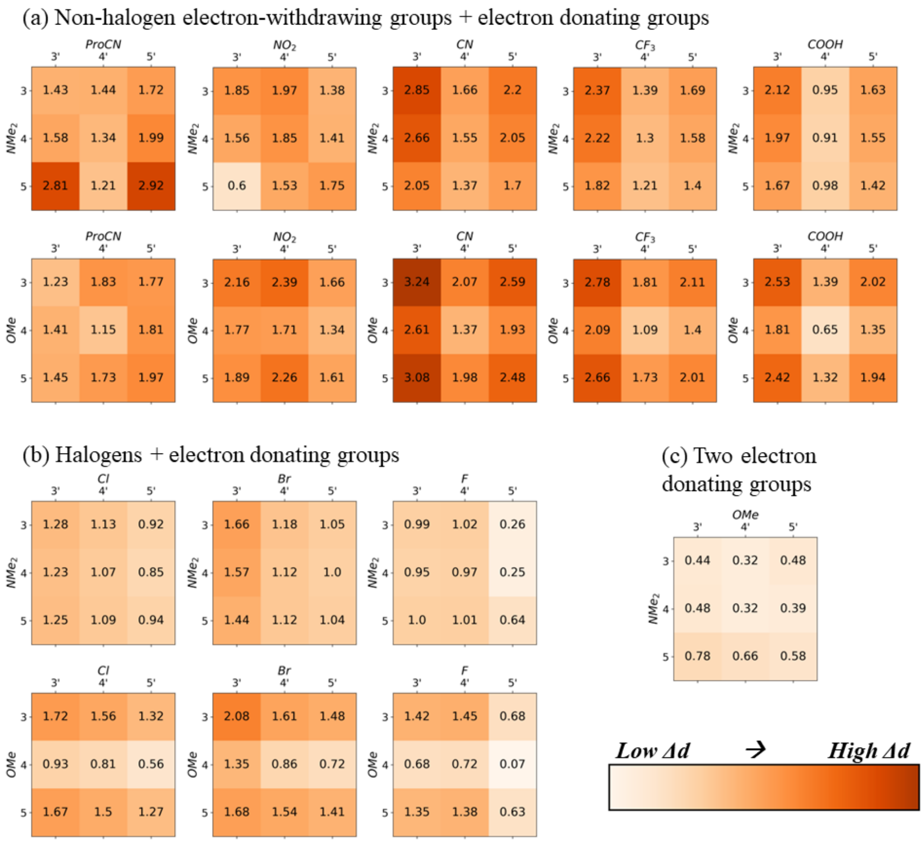

Figure S5: *DFT-calculated Δd, in Debye, for all opposite-side substituent pairs. Darker orange indicates a larger magnitude Δd. All calculations were done in implicit water. Darker colors indicate higher values. The color scale is global, and color can be used to compare between subsistent pairs.*

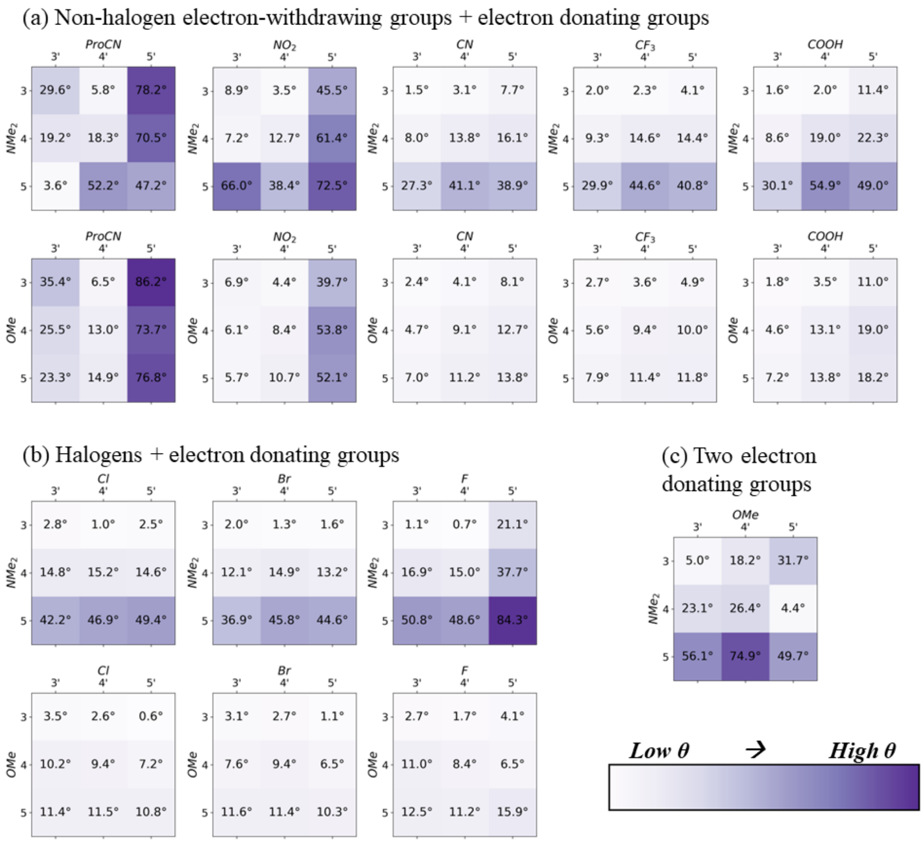

Figure S6: *DFT-calculated* θ *values between μ and Δd, in degrees, for all opposite-side substituent pairs. Darker purple indicates a larger* θ*. All calculations were done in implicit water. Darker colors indicate higher values. The color scale is global, and color can be used to compare between subsistent pairs.*

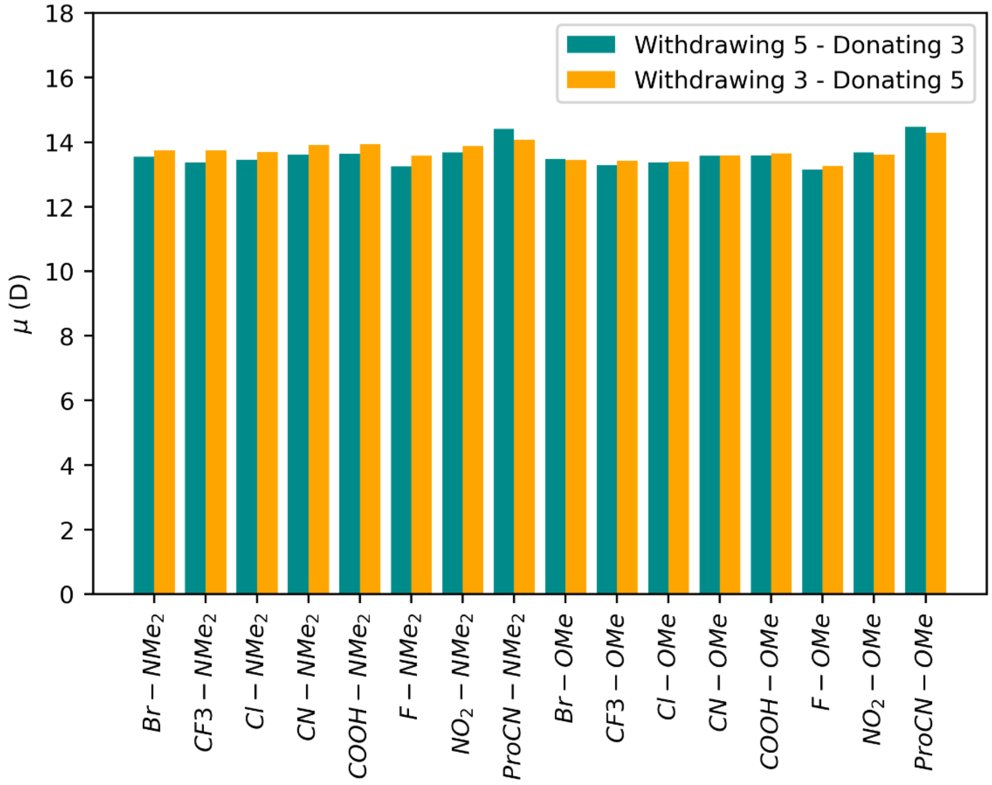

Figure S7: μ values for same-side "push-pull" substituent pairs. "Withdrawing 5 – Donating 3" refers to a configuration where the electron withdrawing group (the first listed substituent) is located on position 5, and the electron donating group (the second listed substituent) is located on position 3. "Withdrawing 3 – Donating 5" refers to a configuration where the electron withdrawing group is on position 3, and the electron donating group is placed on position 5. All calculations were performed in vacuum.

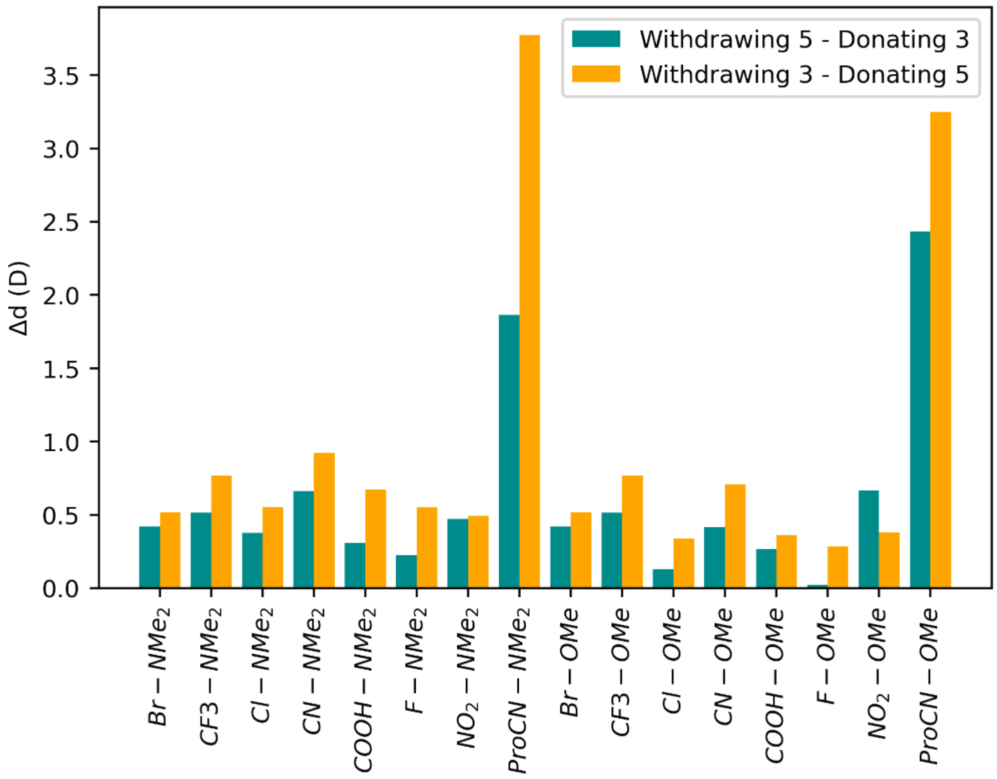

Figure S8: Δd values for same-side substituent pairs. "Withdrawing 5 – Donating 3" refers to a configuration where the electron withdrawing group (the first listed substituent) is located on position 5, and the electron donating group (the second listed substituent) is located on position 3. "Withdrawing 3 – Donating 5" refers to a configuration where the electron withdrawing group is on position 3, and the electron donating group is placed on position 5. All calculations done in vacuum.

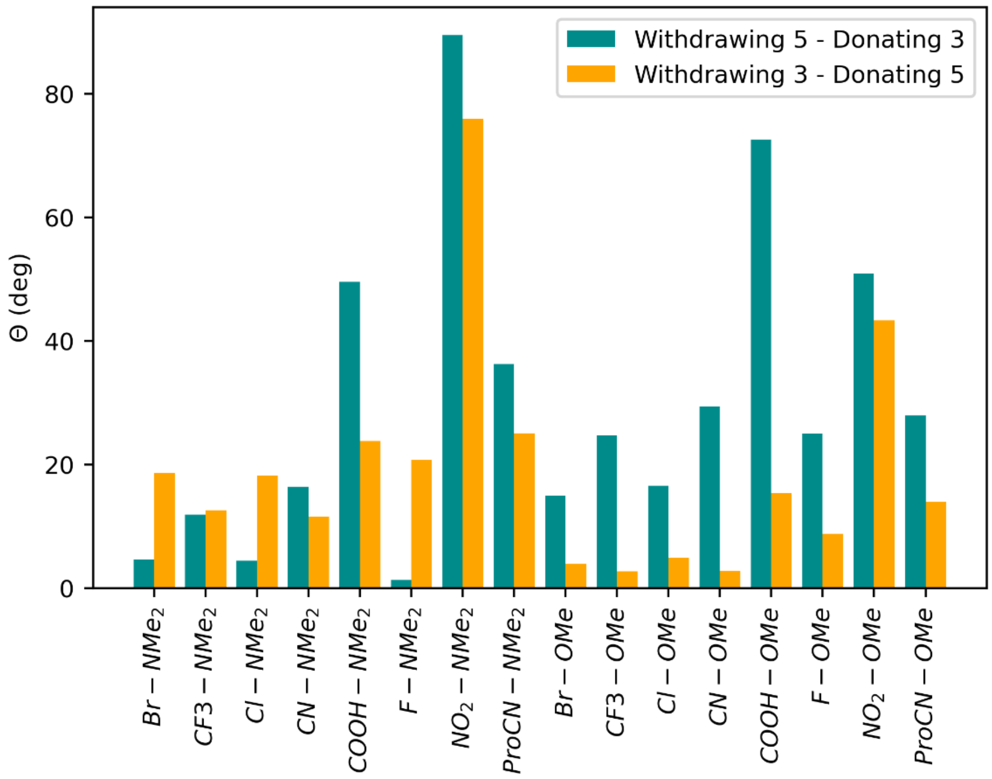

Figure S9: ϑ values for all same-side substituent pairs. "Withdrawing 5 – Donating 3" refers to a configuration where the electron withdrawing group (the first listed substituent) is located on position 5, and the electron donating group (the second listed substituent) is located on position 3. "Withdrawing 3 – Donating 5" refers to a configuration where the electron withdrawing group is on position 3, and the electron donating group is placed on position 5. All calculations done in vacuum.

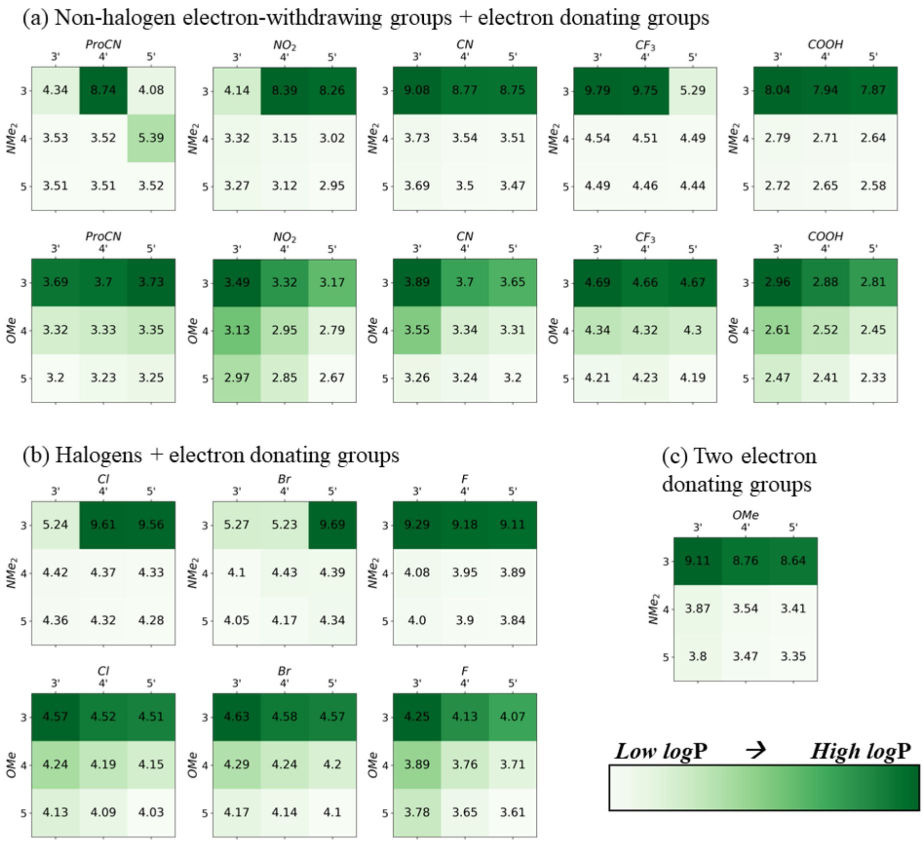

Figure S10: DFT-calculated logP values for all opposite-side substituent pairs. The molecular geometry was optimized in each solvent of interest. Darker green indicates a larger logP. The color range is only consistent for each substituent pair, and color-coding should not be used to compare values between pairs.

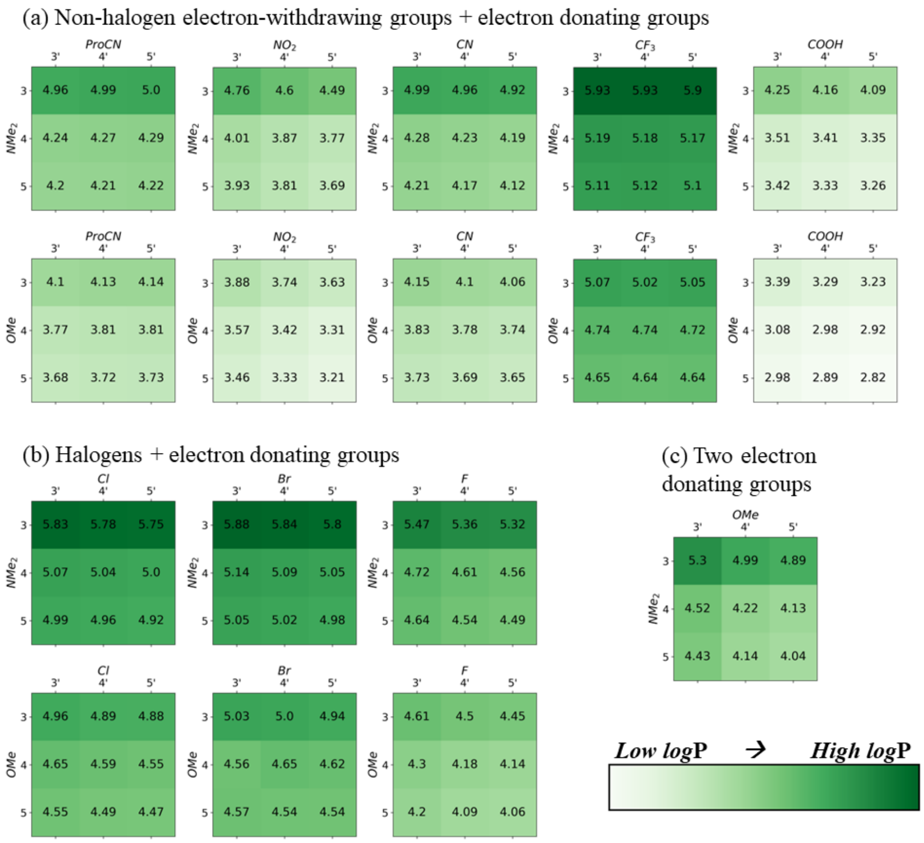

Figure S11. DFT-calculated logP for all opposite-side substituent pairs. Darker green indicates a larger logP. The ground-state geometry, optimized in vacuum, was used for all calculations. Darker colors indicate higher values. The color range is only consistent for each substituent pair, and color-coding should not be used to compare values between pairs.
